# Health related quality of life and psychosocial function among patients with carcinoid tumours. A longitudinal, prospective, and comparative study

**DOI:** 10.1186/1477-7525-5-18

**Published:** 2007-04-11

**Authors:** Camilla Fröjd, Gunnel Larsson, Claudia Lampic, Louise von Essen

**Affiliations:** 1 Department of Public Health and Caring Sciences, Psychosocial oncology, Uppsala University, Uppsala, Sweden; 2 Department of Medical Sciences, Uppsala University, Uppsala, Sweden; 3 Department of Public Health and Caring Sciences, Caring Sciences, Uppsala University, Uppsala, Sweden; 4 Department of Caring Sciences and Sociology, Gävle University, Sweden

## Abstract

**Background:**

The aim was to investigate HRQoL and psychosocial function among patients with carcinoid tumours, longitudinally and prospectively, and to compare HRQoL among patients with carcinoid tumours to that of the Swedish general population. The aim was also to investigate the prevalence of distress during the first year after diagnosis.

**Methods:**

At four assessments during the first year after diagnosis, HRQoL was measured by the EORTC QLQ-C30 3.0, anxiety and depression by the HADS, and prevalence, and worst aspects of distress by an interview guide. ANOVA was performed in order to study changes over time with regard to HRQoL, anxiety and depression. Comparisons regarding HRQoL between patients and the Swedish population were made by the use of one-sample t-tests and changes over time regarding the prevalence of distress was investigated by means of Cochran's Q.

**Results:**

High levels of physical-, emotional-, cognitive-, and social function and somewhat lower levels of role function and global quality of life were reported at all assessments. Role- and emotional function increased over time. Patients reported lower role function and global quality of life and more problems with fatigue and diarrhoea than the Swedish general population, at all assessments. Fatigue, limitations to work and pursue daily activities, and worry that the illness will get worse were among the most prevalent aspects at all assessments. At all assessments the majority reported worrying about the family's situation, the ability to care for the family, and worrying before the check-up.

**Conclusion:**

It is concluded that HRQoL and psychosocial function among patients with carcinoid tumours remains stable during the first year, that the patients report a lower HRQoL than the Swedish general population, and that a majority of the patients report a number of aspects of emotional distress. In the clinical care, it should be considered that the majority of patients report not only fatigue and diarrhoea but also worries about their prognosis, their families, tests, and examinations. Efforts to reduce these worries should be made.

## Background

Carcinoid tumours are slow-growing malignancies belonging to the family of neuroendocrine tumours, the incidence is 2.5 in 100 000 [1]. The most common type is midgut carcinoid tumours with a 5-year survival rate of 61% [1]. Neuroendocrine tumours can be divided into functioning and non functioning tumours. Functioning tumours cause symptoms related to an overproduction of hormones while non-functioning tumours produce peptides that do not cause symptoms [1]. Symptoms caused by functioning tumours are flush, diarrhoea, bronchial constriction, and right heart failure caused by overproduction of varying hormones. Abdominal pain due to the tumour itself, lymph node or liver metastases may occur [1]. Surgery is the only treatment that can cure the patients. Some, but not all benefit from chemotherapy. The management usually includes treatment with biological agents such as somatostatin analogues and interferon-α [1]. Both agents control the disease but do not provide a cure. The aims of treatment are to reduce hormone levels, control hormonal symptoms, prevent further tumour growth, and possibly also tumour reduction [1].

Common adverse effects of somatostatin analogues are nausea, abdominal cramps, loose stools, mild steathorrea, and flatulence. These symptoms usually decrease within a few weeks. Flu-like symptoms are very common, usually short lasting, consequences of interferon treatment. Chronic fatigue and mild depression may develop in approximately 50% of the patients treated with interferon [2]. The treatment should be carefully monitored by a complete history and physical examination approximately every 3 months [2]. Biochemical parameters (tumour markers) are tested every 3 to 6 months and the patients should be examined using conventional imaging studies (CT, MRI, ultrasonography, octreoscan) every 6 months [2,3].

It has been reported that patients with carcinoid tumours enjoy a good health related quality of life (HRQoL) and low levels of anxiety and depression, in early as well as later stages of the disease [4-6]. However, the findings of a longitudinal, prospective study in which 24 patients with carcinoid tumours were included indicate that they, during the first year of treatment with somatostatin analogues and/or interferon-α, report a lower emotional- and role function, lower global quality of life and more problems with fatigue, nausea/vomiting, appetite loss, and diarrhoea compared to the Swedish general population [6].

Since carcinoid tumours may be presented with problems typical for the disease it was questioned whether the EORTC QLQ-C30 and Hospital Anxiety and Depression Scale, previously used to measure patients' with carcinoid tumours psychosocial function [4-6], included all problems that may be of relevance for the patients. Therefore, an interview study with patients with carcinoid tumours, and staff caring for them was undertaken [7]. The findings indicate that the patients report a number of physical, social, and emotional aspects of distress [7]. Pain and flush are examples of aspects of physical distress [7] whereas limited possibilities to work/pursue daily activities are examples of aspects of social distress and worry that the disease will get worse an example of emotional distress [7]. Several of the identified aspects of distress are not included in the EORTC QLQ-C30 [8] or the Hospital Anxiety and Depression Scale [9]. In the studies [4-7] referred to above, the time the patients had been living with their disease varied from 1 to 96 months which limits the possibilities to make conclusions regarding the patients' HRQoL, and levels of anxiety and depression, at certain time-points during the course of the disease. This circumstance, together with the fact that the prevalence of disease-related aspects of distress among patients with carcinoid tumours has not been investigated indicates that there is room for more knowledge about how patients with carcinoid tumours experience their life situation.

The present study sets out to add to knowledge about HRQoL and psychosocial function among patients with carcinoid tumours by following a group of individuals over time, starting shortly after diagnosis. The following research questions were posed: 1. Do patient ratings of HRQoL, anxiety, and depression change over time from diagnosis?; 2. How do patients rate their HRQoL compared to the Swedish general population shortly after each of the first four admissions to hospital?; 3. What is the prevalence of physical, social, and emotional aspects of distress shortly after each of the first four admissions to hospital?; 4. Which physical, social, and emotional aspects of distress are reported as the worst shortly after each of the first four admissions to hospital? and 5. Does the prevalence of physical, social, and emotional aspects of distress change over time from diagnosis?

## Methods

### Design

This study is based on a longitudinal, prospective design with four assessments (T1–T4). Each of these occurred shortly after each of the patients' first four admissions to the Department of Endocrine Oncology (DepEO), Uppsala University Hospital, Uppsala, Sweden.

### Sample and setting

To be eligible patients should be referred to the DepEO for the first time, age ≥18 years, have a good command of the Swedish language, and not be cognitively impaired. During the inclusion period (April 2001–August 2003) 103 patients with a suspected diagnosis of carcinoid were referred to the DepEO. Five of these were not asked about participation due to administrative reasons, of the remaining patients 76 accepted participation. At T1, 17 patients were excluded for the following reasons: 7 were not diagnosed with carcinoid, 6 would not be further referred to the DepEO, and 4 were too ill to participate. Thus, 59 patients were included in the study at T1, 36 of these participated at all assessments (T1–T4). Data from these patients are reported in this study and their medical and demographic characteristics are presented in Table 1. For a presentation of reasons for attrition at T2–T4, see Table 2. The patients in the study were referred to the DepEO from several hospitals in Sweden, for a diagnosis or a verification of a suspected diagnosis of carcinoid tumours. The patients participating in the study were all in-patients, the mean duration of each hospital admission was 6 days (SD = 2) at the first, 4 days (SD = 1) at the second, 3 days (SD = 1) at the third, and 3 days (SD = 0.7) at the fourth admission. During these admissions, patients' biochemical parameters (tumour markers) could be tested, and patients could be subjected to conventional imaging studies such as CT, MRI, ultrasonography, octreoscan [2,3] and in some cases patients received treatment (see Table 1). The mean time between the first and the second admission was 3 months (SD = 1.3), 4 months (SD = 1.4) between the second and the third, and 5 months (SD = 1.7) between the third and the fourth admission.

**Table 1:** Background data at T1 for patients (N = 36). All figures represent number of patients unless stated

	**T1**	**T2**	**T3**	**T4**
Male	19 (53%)			
Female	17 (47%)			
Mean age (SD)	60 (10%)			
Married/cohabiting	25 (70%)			
Working	7 (19%)			
On sick leave	14 (39%)			
Old age pensioner	15 (42%)			
Metastatic	25(70%)	28 (78%)	28 (78%)	28 (78%)
Levels of Chromogranin A, nmol/liter (ref < 4)	37	42	53	76
Levels of U-5HIAA, μmol/h (ref < 2.1)	13	12	23	14
Mean time since surgery, months^1,2 ^(SD)	3.1 (2.5)			
Interferon	5 (14%)	8 (22%)	6 (27%)	4 (11%)
Octreotid	5 (14%)	6 (27%)	6 (27%)	6 (27%)
Interferon and Octreotid	6 (27%)	10 (28%)	12 (33%)	9 (25%)
Other (Chemotherapy)	4 (11%)	5 (14%)	6 (27%)	3 (<1%)
Octreotid and chemotherapy	-	-	-	1 (<1%)
No treatment	16 (44%)	7 (19%)	6 (27%)	13 (36%)

^1 ^14 patients had surgery prior to the first admission to the Department of Endocrine Oncology.

^2 ^3 patients had surgery between the first and second admission to the Department of Endocrine Oncology.

**Table 2:** Reasons for attrition at T2–T4

**Reason for attrition**	**T2**	**T3**	**T4**
Withdrawal of consent	5	3	1
Patients would not be further referred to the Department of Endocrine Oncology	4	2	2
Death	-	1	3
Too ill to participate	1	-	1
Total	10	6	7

### Procedure

Ethical approval was obtained from the local ethics committee at the faculty of Medicine at Uppsala University. The first author (CF) provided potential participants with oral and written information about the aim and procedure of the study at their first admission to the DepEO. They were informed that participation was voluntary, that confidentiality was guaranteed, and that neither participation, non-participation or withdrawal would affect their treatment and care. Shortly thereafter CF asked patients about consent. Patients who accepted participation and were eligible at T1, T2, T3, and T4 received the instruments (see Instruments) at the preceding admission to the DepEO. Patients were asked to complete these at home, after their hospital-stay, and a time when the patient should be asked to provide his/her responses via telephone, was agreed upon with CF. Data was collected from one week up to one month after each admission. Medical and demographic data were collected from the medical journals by CF.

### Instruments

#### HRQoL

HRQoL was measured with the EORTC QLQ-C30 3.0 [8] which includes 30 questions incorporated in five functional scales: physical (PF), role (RF), cognitive (CF), emotional (EF), and social (SF), three symptom scales: fatigue (FA), pain (PA), and nausea and vomiting (NV), and a global health and quality of life scale (QL). Also included are single items assessing: dyspnoea (DY), appetite loss (AP), sleep disturbance (SL), constipation (CO), diarrhoea (DI), and perceived financial impact (FI) of the disease and treatment. Responses were provided on Likert scales, coded 1–4 with the response alternatives not at all, a little, quite a bit, and very much. Responses for global health and quality of life were provided on scales with the end-points 1 (very poor) and 7 (excellent). The scores were transformed in accordance with the EORTC guidelines to 0–100 scores [10]. A higher score on functional scales represents a higher level of function, while a higher score on symptom scales represents a higher level of symptoms. Good reliability and validity has been shown for the instrument [8]. In this study, Cronbach's alpha values for the subscales were >.70 at T1–T4, with the exception of RF (.60 at T2), CF (.35 at T2, .46 at T3, and .67 at T4), NV (.43 at T1, .65 at T3, and impossible to compute alpha at T4). Patients were asked to answer the questions according to the last week.

### Anxiety and depression

Anxiety and depression was measured with the Hospital Anxiety and Depression Scale (HADS) [9]. The instrument consists of two subscales (7 items in each subscale), one measuring anxiety (A) and one depression (D). Responses are provided on verbal scales, coded 0–3. Subscale scores range from 0 to 21. The HADS has proved to be a useful clinical indicator of anxiety and depression [11-13] among patients with somatic illnesses. Cronbach's alpha values at T1–T4 were high for the anxiety (.80–.90) and the depression subscale (.82–.92.). Patients were asked to answer the questions according to the last week.

### Aspects of distress

In a previous study [7] patients and nurses were posed semi structured interview questions about distress related to the disease and treatment. The data was analysed using content analysis (7), and 24 aspects of distress were identified. These aspects were related to 3 dimensions; physical (10 aspects), social (5), and emotional (10) aspects of distress [7] (see Table 5 for a presentation of the dimensions and aspects of distress). By using these aspects of distress as a starting point, an instrument was constructed for the present study, aiming at measuring the prevalence, and worst aspects of distress. The instrument consists of 3 dimensions, a physical (10 aspects), social restrictions (5 aspects), and emotional (9 aspects) dimension (in the present study the aspect worry before illness was omitted from the emotional dimension). For each aspect patients were asked to rate the level of distress, and provide their responses on a Likert scale from 0 (no distress) to 5 (very much distress). Thereafter the patients were asked, by a separate question, to identify which of the aspect that had been the worst, within each dimension. For the dimension social distress, the patients were asked to state which of the 10 aspects of physical distress they perceived had caused social distress. Patients were encouraged to identify only one aspect as worst, within each dimension, but when patients chose to report more than one aspect as being the worst, all reported aspects were included in the presentation of the results. Patients were asked to answer the questions according to the last four weeks.

### Background data

Patients were asked to report age, gender, civil status and occupational status. Data on medical variables such as treatment, metastasis, surgery, and biochemical parameters were gathered through the patients' medical records.

### Data analysis

All statistical analyses were conducted using the Statistical Package for the Social Sciences (SPSS) version 14.0 (SPSS Inc, Chicago, IL). Research question 1 was answered by repeated measures ANOVA whereas research question 2 was answered by one-sample t-test. As suggested by others [14] a difference of more than 10 points between two values for the same variable in EORTC QLQ-C30 was considered as indicating a difference of clinical interest. For the comparison between patient scores on the EORTC QLQ-C30 and corresponding Swedish population norms [15], corrections for age and gender were made. When calculating prevalence of aspects of distress it was assumed that if a patient answered "no distress" in response to a question about an aspect, the same aspect was not prevalent for the same individual. Remaining response alternatives were assumed to indicate that the aspect, at least to some extent, was experienced as distressing and thus prevalent. Research question 5 was answered by Cochran's Q.

Missing values for the EORTC QLQ C-30 were replaced according to the manual for the EORTC QLQ C-30 [10]. After replacing missing values the internal dropout for EORTC QLQ C-30 was <2% at T2. Missing values for the HADS were replaced by the mean values of the patient's answers to the remaining items of the respective subscale, provided that at least four of the items in the subscale had been answered. Missing values for the interview guide were not replaced. The internal dropout for aspects of physical distress was 0.5–3% at T1–T4, social distress 0–3% at T1–T4, and emotional distress 0–4% at T1–T4.

A significance level of p < .01 was chosen for all statistical analyses.

## Results

See Table 1 for a presentation of demographic and medical background data for the patients participating in the study.

### HRQoL, anxiety, and depression

Mean values and standard deviations for HRQoL, anxiety, and depression at T1–T4 are presented in Table 3. The mean values for role function and global quality of life were somewhat lower than for the remaining scales at all assessments. At all assessments the highest level of problems were reported for fatigue followed by pain, dyspnoea, and diarrhoea. Low mean values were reported for anxiety and depression at all assessments.

**Table 3:** Mean values (M) and standard deviations (SD) for the EORTC QLQ-C30 scales and single items and the HADS subscales at (T1–T4) (n = 36)

Scales/single items	**T1**	**T2**	**T3**	**T4**
	M, SD	M, SD	M, SD	M, SD
PF^a^	80 (22)	80 (20)	81 (22)	79 (22)
RF^a^	53 (39)	64 (31)	69 (28)	69 (35)
EF^a^	77 (20)	75 (23)	75 (21)	82 (19)
CF^a^	85 (21)	85 (17)	83 (18)	84 (18)
SF^a^	77 (26)	80 (28)	80 (26)	79 (27)
QL^a^	58 (19)	61 (23)	58 (24)	58 (25)
FA^b^	35 (26)	38 (27)	35 (24)	34 (24)
NV^b^	3 (8)	10 (20)	6 (12)	7 (12)
PA^b^	20 (28)	23 (31)	27 (33)	21 (27)
DY^b^	27 (31)	22 (24)	30 (36)	29 (34)
SL^b^	18 (24)	11 (18)	13 (18)	18 (26)
AP^b^	10 (19)	18 (28)	8 (17)	13 (24)
CO^b^	9 (22)	13 (22)	10 (21)	9 (23)
DI^b^	25 (35)	21 (29)	23 (31)	30 (27)
FI^b^	14 (23)	15 (26)	12 (23)	14 (26)
A^c^	5.1 (4.1)	5.1 (4.7)	4.4 (3.9)	4.1 (3.4)
D^c^	4.1 (3.7)	4.6 (4.6)	5.0 (4.0)	4.5 (3.6)

PF = Physical function, RF = Role function, CF = Cognitive function,

EF = Emotional function, SF = Social function, QL = Global quality of life,

FA = Fatigue, NV = Nausea/vomiting, P = Pain, DY = Dyspnoea,

SL = Sleep disturbances, AP = Appetite loss, CO = Constipation,

DI = Diarrhoea, FI = Perceived financial impact, A = Anxiety, D = Depression

^a ^Scores range from 0 to 100, a higher score represents a higher level of function.

^b ^Scores range from 0 to 100, a higher score represents a higher level of symptoms.

^c ^Scores range from 0–21, a higher score represents a higher level of problems.

Patients reported lower emotional function at T3 compared to T4 [F(3, 102) = 3.50; p < .01].

The mean value for role function increased more than 10 points, i.e. of clinical interest, from T1 to T2, T3, and to T4.

See Table 4 for a presentation of the observed scores and population norms for HRQoL at T1–T4. The difference between scores was larger than expected by chance for role function at T1–T3 (t = 3.26–4.99, df = 34–35, p < .01, .001), social function at T1 (t = 3.17, df = 35, p < .01), and global quality of life at T1–T4 (t = 3.74–5.75, df = 34–35, p < .01, .001). The observed score was more than 10 points below the population norms for role- and social function, and global quality of life at all assessments and at all assessments, the observed score was more than 10 points above the population norms for fatigue and diarrhoea. All these differences were larger than expected by chance (t = 3.16–5.71, df = 34–35, p < .01, .001). At T2 the observed score was more than 10 points higher than the population norms for appetite loss (t = 2.93, df = 34, p < .01), and at T3 and T4 the observed score was more than 10 points higher than the population norms for dyspnoea.

**Table 4:** Mean (M) for observed scores (O) and population norms (P) and differences (D) between the observed scores and population norms for the EORTC QLQ C-30 scales and single items at T1–T4

	**T1**			**T2**			**T3**			**T4**		
	O	P	D	O	P	D	O	P	D	O	P	D
	M	M	D	M	M	D	M	M	D	M	M	D
PF^a^	80	87	-7	80	87	-7	81	87	-6	79	87	-8
RF^a^	53	85	-32**	64	85	-21**	69	85	-16*	69	85	-16
EF^a^	77	83	-6	75	83	-8	75	83	-8	82	83	-1
CF^a^	85	88	-3	85	88	-3	83	88	-5	84	88	-4
SF^a^	77	91	-14*	80	91	-11	80	91	-11	79	91	-12
QL^a^	58	76	-18**	61	76	-15**	58	76	-18**	58	76	-18**
FA^b^	35	21	14*	38	21	17**	35	21	14**	34	21	13*
NV^b^	3	3	0	10	3	7	6	3	3	7	3	4
P^b^	20	20	0	23	20	3	27	20	7	21	20	1
DY^b^	27	17	10	22	17	5	30	17	13	29	17	12
SL^b^	18	19	-1	11	19	-8	13	19	-6	18	19	-1
AP^b^	10	4	6	18	4	14*	8	4	4	13	4	9
CO^b^	9	6	3	13	6	7	10	6	4	9	6	3
DI^b^	25	5	20*	21	5	16*	23	5	18*	30	5	25**
FI^b^	14	8	6	15	8	7	12	8	4	14	8	6

PF = Physical function, RF = Role function, CF = Cognitive function, EF = Emotional function, SF = Social function, QL = Global quality of life, FA = Fatigue, NV = Nausea/vomiting, P = Pain, DY = Dyspnoea, SL = Sleep disturbances, AP = Appetite loss, CO = Constipation, DI = Diarrhoea, FI = Perceived financial impact, A = Anxiety, D = Depression

^a ^Scores range from 0 to 100, a higher score represents a higher level of function.

^b ^Scores range from 0 to 100, a higher score represents a higher level of symptoms.

* p < .01, ** p < .001

**Table 5:** Prevalence and worst aspects of physical, social, and emotional distress at T1–T4 (n = 36)

	**T1**		**T2**		**T3**		**T4**	
	Prevalence	Worst^1^	Prevalence	Worst^1^	Prevalence	Worst^1^	Prevalence	Worst^1^
**Physical distress**								
Fatigue	25 (69%)	11	31 (86%)	15	32 (89%)	12	30 (83%)	17
Diarrhoea	18 (50%)	10	20 (56%)	10	22 (61%)	11	25 (69%)	13
Dry skin/mucous membranes	14 (39%)	2	22 (61%)	5	22 (61%)	3	25 (69%)	6
Pain-stomach	18 (50%)	4	20 (56%)	3	20 (56%)	8	23 (64%)	4
Dyspnoea	18 (50%)	3	21 (58%)	5	20 (56%)	8	21 (58%)	10
Flush	19 (53%)	8	21 (58%)	1	21 (58%)	5	11 (30%)	1
Appetite loss	14 (39%)	1	17 (47%)	5	21 (58%)	4	15 (42%)	4
Pain-muscles	15 (42%)	1	17 (47%)	6	15 (42%)	5	18 (50%)	6
Nausea	14 (39%)	1	15 (42%)	3	19 (53%)	5	17 (47%)	6
Insomnia	13 (36%)	4	15 (42%)	3	17 (47%)	1	18 (50%)	5
**Social distress**								13
Work/pursue daily activities	23 (64%)	15	24 (67%)	15	24 (67%)	13	26 (72%)	6
Perform physical activities	23 (64%)	7	23 (64%)	3	25 (69%)	10	27 (75%)	6
To travel	20 (56%)	2	17 (47%)	5	23 (64%)	8	21 (58%)	2
Dine out/go to theatre etc	17 (47%)	-	18 (50%)	3	19 (53%)	6	19 (53%)	6
Associate with friends	17 (47%)	3	17 (47%)	3	20 (56%)	6	18 (50%)	
**Emotional distress**								
Worry that the illness will get worse	33 (92%)	20	32 (89%)	10	30 (83%)	16	31 (86%)	16
Depression	32 (89%)	1	27 (75%)	5	29 (80%)	9	26 (72%)	10
Irritation	27 (75%)	5	26 (72%)	5	29 (80%)	6	27 (75%)	6
Worry that the family can not cope with the illness	29 (80%)	6	29 (80%)	4	25 (69%)	5	23 (64%)	7
Worry before check-up	27 (75%)	4	25 (69%)	3	24 (67%)	7	26 (72%)	7
Worry that the illness will interfere with ability to care for the family	25 (69%)	5	21 (58%)	3	24 (67%)	5	22 (61%)	1
Troublesome tests/examinations	25 (69%)	4	17 (47%)	4	20 (56%)	1	24 (67%)	2
Bother by changed appearance	7 (19%)	-	14 (39%)	1	18 (50%)	2	17 (47%)	3
Bother by changed sexual activity	13 (36%)	-	14 (39%)	-	16 (44%)	1	19 (53%)	2

^1 ^Some patients reported more than one aspect of distress as being worst.

### Aspects of distress

See Table 5 for a presentation of prevalence and worst aspects of distress at T1–T4. Fatigue was the most prevalent, and flush, pain-stomach, dyspnoea, dry skin/mucous membranes, diarrhoea, and appetite loss were among the most prevalent aspects of physical distress at all assessments. At all assessments fatigue and diarrhoea were reported as the worst aspects of physical distress by most patients. A difference over time with regard to the prevalence of flush (Q (3) 13.2, p < .005), with a lower prevalence at T4 than at T1–T3, and dry skin/mucous membranes (Q (3) 12.7, p < .005), with a higher prevalence at T4 than at T1 was demonstrated.

Limited possibilities to work/pursue daily activities and to perform physical activities were the most prevalent aspects of social distress at all assessments. At T1–T3 limited possibilities to work/pursue daily activities was reported as the worst aspect of social distress by most patients. At T4 an equal number of patients reported this aspect and limited possibilities to perform physical activities and to dine out/go to theatre as the worst aspect of social distress. Fatigue followed by diarrhoea and dyspnoea were reported most frequently as the aspects causing social distress at all assessments.

Worry that the illness will get worse was the most prevalent, and depression, irritation, worry that the family cannot cope with the illness, and worry before check-up were among the most prevalent emotional aspects of distress at the respective assessment. At all assessments worry that the illness will get worse was reported as the worst aspect of emotional distress by the majority of the patients. A difference over time with regard to the prevalence of bother by changed appearance (Q (3) 12.0, p < .01) with a higher prevalence at later assessments than at T1 was demonstrated.

When looking at the prevalence of all aspects of distress together, worry that the illness will get worse was the most prevalent aspect followed by depression, worry that the family cannot cope with the illness, irritation, and worry before check-up at T1. At T2 the corresponding aspects were worry that the illness will get worse, fatigue, worry that the family can not cope with the illness, depression, and irritation, at T3 fatigue, worry that the illness will get worse, depression, irritation, limited possibility to perform physical activities, and worry that the family can not cope with the illness, and at T4 worry that the illness will get worse, fatigue, limited possibility to perform physical activities, irritation, worry before check-up, and limited possibility to work/pursue daily activities. Most of these aspects are of an emotional nature.

When taking together all aspects of distress that patients reported as being the worst, worry that the illness will get worse, fatigue, and limited possibility to work/pursue daily activities were the aspects considered the overall worst by most patients at T1–T3, when looking at the three dimensions together. At T4 the corresponding aspects were: fatigue, worry that the illness will get worse, and diarrhoea.

## Discussion

At all assessments patients reported high levels of physical-, emotional-, cognitive-, and social function however somewhat lower levels of role function and global quality of life. Most problems were reported with fatigue, dyspnoea, diarrhoea, and pain. The results support previous results and taking the present results into consideration, together with previous results, [5,6,16] it may be concluded that patients with carcinoid tumours enjoy good HRQoL from the early stages of the disease as well as later on. Patients' role- and emotional function increased over time. Apart from these changes patients' HRQoL and psychosocial function did not change during the first year after diagnosis. On one hand it could be expected that once patients receive their diagnosis and treatment aiming at reducing the hormone-related symptoms is initiated, patients' HRQoL and psychosocial function should increase. On the other hand it could be expected that patients' HRQoL and psychosocial function, as a consequence of the illness itself and disruption of the previous normal life, should decrease over time. However, the results do not support any of these expectations. The lack of significant and/or clinically relevant changes could also be explained by methodological circumstances such as a small sample and inappropriate instruments and/or by, if it is assumed that HRQoL and psychosocial function ought to deteriorate over time, an adaptation to a difficult life situation.

When comparing the patients' HRQoL to the Swedish population norms, it is revealed that the patients' HRQoL is significantly lower in a statistical sense than that reported by the Swedish population for fatigue, diarrhoea, and overall quality of life at all assessments and for role function at the first three assessments. The findings support previous results [6]. In this study clinical significance, in most cases, coincides with statistical significance which highlights that identified differences between patients with carcinoid tumours and the Swedish population are of clinical relevance.

Some of the patients in the present study were treated with interferon and it is known that interferon may cause fatigue and mild depression [2]. In this study we found it difficult to analyze potential differences in HRQoL and psychosocial function between patients treated with interferon versus patients treated with chemotherapy, octreotide or no treatment at all, due to small sub-groups.

The sample may be considered small from a statistical point of view. However, when considering the size of the sample it should be remembered that carcinoid tumours are very rare. The patients were consecutively included during approximately two and a half years at the Department of Endocrine Oncology, Uppsala University Hospital, at the time of the study the main centre in Sweden specialized in treating endocrine tumours. In addition, the sample is unique since it is homogenous with regard to time since diagnosis and the timing of the assessments.

The present study is to the best of our knowledge one of only a few studies in which patients with carcinoid tumours have been asked to rate their HRQoL, anxiety, and depression repeatedly after diagnosis. When considering the generalizability of the findings it is important to consider the number of non-participants and drop-outs and whether these persons differ from the participants. It is possible that if health should deteriorate, patients may choose to withdraw. However, analyses investigating whether the participating patients' HRQoL and psychosocial function differed from that of the dropouts at T1 did not show any differences.

This study investigated the prevalence of some inductively derived aspects of physical, social, and emotional distress previously found to be of relevance for patients with carcinoid tumours [7]. By presenting the prevalence of these aspects and which of these that were considered to be the worst, the psychosocial function of patients with carcinoid tumours was further illuminated. The findings demonstrate that fatigue was the most prevalent and diarrhoea among the four most prevalent aspects of physical distress at all assessments as well as considered the worst aspects of physical distress by most patients at all assessments. The findings with regard to physical aspects of distress correspond with the EORTC QLQ C-30 data as these demonstrate at least high levels of problems with fatigue and diarrhoea at all assessments and a higher level of problem with these concerns than that for the Swedish general population at all assessments.

Limitations with regard to pursuing work, and daily- and physical activities were the most prevalent aspects of social distress at all assessments and limitations with regard to work and daily activities was considered the worst of these aspects by most patients at all assessments. Fatigue and diarrhoea were, at all assessments, reported as the most frequent reasons for social restrictions, which underscores the importance of realizing the important consequences of these problems.

Worry that the illness will get worse was the most prevalent as well as considered the worst aspect of emotional distress by most respondents at all assessments. The results show that the majority of the patients, at all assessments, worried, at least to some extent, about how their families would cope with the disease and about whether they would be able to care for their families in spite of the disease. A substantial number of the patients also reported worrying before troublesome tests and examinations and about what the test results would show. None of these aspects of emotional distress are included in the EORTC QLQ-C30, at the time of the study, the HRQoL instrument that previously had been used when investigating HRQoL among patients with endocrine gastrointestinal tumours [5,6,16]. A majority of the patients worry about a number of disease- and treatment related concerns at all assessments as well as report feeling depressed and irritated at all assessments.

Although the reported prevalence of physical, social, and emotional distress does not provide knowledge about the significance of the aspects of distress, the results illustrates that there are several aspects of emotional distress likely to be of concern for the patients. While the EORTC QLQ C-30, and especially the new carcinoid module [17] well covers the physical aspects of the carcinoid disease, and the HADS cover the levels of anxiety and depression, many concerns of a more emotional matter may be unrecognized by doctors who care for these patients. Thus, in this study, sensitivity has been prioritized over specificity, as it is our belief that it is important to be aware of what problems patients with carcinoid tumours may be presented with, and which of these problems patients report as being the worst. Further, these findings with regard to aspects of distress give another impression concerning the psychosocial function of patients' with carcinoid tumours than those from the EORTC QLQ-C30 which indicate that the patients enjoy good psychosocial function at all assessments. However, on the basis of the findings from this study it cannot be concluded which picture that best illustrates the life situation of these patients – they do however illustrate that patients with carcinoid tumours may perceive certain disease- and treatment related aspects not included in the EORTC QLQ-C30 as troublesome. The EORTC HRQoL-group has recently developed a disease-specific module for patients with gastrointestinal neuroendocrine tumours [17]. In this module, some aspects of emotional distress identified in this study, for instance concerning family life and worry about results from tests and examinations are included.

## Conclusion and implications

It is concluded that HRQoL and psychosocial function among patients with carcinoid tumours remains stable during the first year after diagnosis, that the patients report a lower HRQoL than the Swedish general population, and that a majority of the patients report a number of aspects of emotional distress. In the clinical care of these patients, in addition to paying attention to the physical problems such as diarrhoea and fatigue it should also be considered that the majority of the patients also worry about their prognosis, their families, tests, and examinations. Efforts to reduce these worries should be made.

## Competing interests

The author(s) declare that they have no competing interests.

## Authors' contributions

LvE designed the study and assisted in the data analysis and the critical revision of the manuscript. CF carried out the data collection, analysed the data and wrote the manuscript. CL assisted in the critical revision of the manuscript. GL assisted in the data analysis, and critical revision of the manuscript.
